# From Parasite to Therapy: Immunomodulatory Effects of a *Trichuris trichiura* Derived Recombinant Protein in Allergic Airway Disease

**DOI:** 10.1111/cea.70374

**Published:** 2026-06-21

**Authors:** Jessica Cristiane da Conceição de Andrade, Eduardo Santos da Silva, Jennifer Emily Anunciação Sousa, Jade Liz Ferreira Mendes Souza, Carolina Melo Orrico‐Ferreira, Lorena Miranda Souza, Raphael Chagas Silva, Silvia Letícia Bispo dos Santos, Antônio Márcio Santana Fernandes, João Vitor Borges Rios, Paulo Emílio Oliveira Cruz, Vítor Lima Miranda Melo, Emília Maria Medeiros de Andrade Belitardo, Luís Gustavo Carvalho Pacheco, Carina Silva Pinheiro

**Affiliations:** ^1^ Laboratory of Allergology and Acarology, Institute of Health Sciences Federal University of Bahia Salvador Brazil; ^2^ Post Graduate Program of Immunology Federal University of Bahia Salvador Brazil; ^3^ Gonçalo Moniz Institute, Oswaldo Cruz Foundation (Fiocruz) Salvador Brazil; ^4^ Instituto de Investigação em Doenças Infecciosas e Crônicas das Mucosas e Pele (INCT Mucosa e Pele) Belo Horizonte Brazil

## Abstract

• C4299 reduced airway Th2 cytokines and enhanced regulatory mediators in allergic mice.

• C4299 expanded antigen‐responsive Tregs and shifted antibody responses towards IgG2a.

To the Editor,

Helminth‐derived molecules have attracted considerable interest as a potential source of therapeutic biomolecules for allergic diseases due to their ability in inducing regulatory immune responses and counterbalance type 2 inflammation [1, 2]. Helminths have co‐evolved with the human immune system and can promote immune tolerance through regulatory cytokines such as IL‐10 and TGF‐β and the expansion of regulatory T cells [3]. These properties have inspired the search for parasite‐derived molecules that could reproduce such immunomodulatory effects without infection.

*Trichuris trichiura*, a soil‐transmitted helminth infecting nearly 500 million people worldwide, represents a potential source of immunomodulatory proteins [2]. Proteomic and transcriptomic analyses of this parasite have identified several candidate molecules with regulatory properties [2, 4]. Among them, C4299 protein has been previously produced in its recombinant form and displayed the ability to induce IL‐10 production in human peripheral blood mononuclear cells, suggesting tolerogenic potential [2]. However, its immunomodulatory activity in vivo has not yet been explored. In this study, we investigated the in vitro immunomodulatory effects of the recombinant C4299 on THP‐1‐derived macrophages, alongside its in vivo impact in a murine model of allergic airway inflammation induced by the mite *Blomia tropicalis*, a primary allergen source in tropical regions [5, 6, 7].

Stimulation with recombinant C4299 did not compromise THP‐1‐derived macrophages' viability and induced secretion of IL‐1β, IL‐6, IL‐10 and IFN‐γ (Figure 1A). The induction of IL‐10 was particularly notable given its central role in immune tolerance and allergen immunotherapy [3, 5, 8]. These findings demonstrate that C4299 is biologically active and capable of stimulating innate immune responses towards a regulatory profile, consistent with similar outcomes observed in our previous study with peripheral blood mononuclear cells from mite‐allergic individuals [2].

**FIGURE 1:**
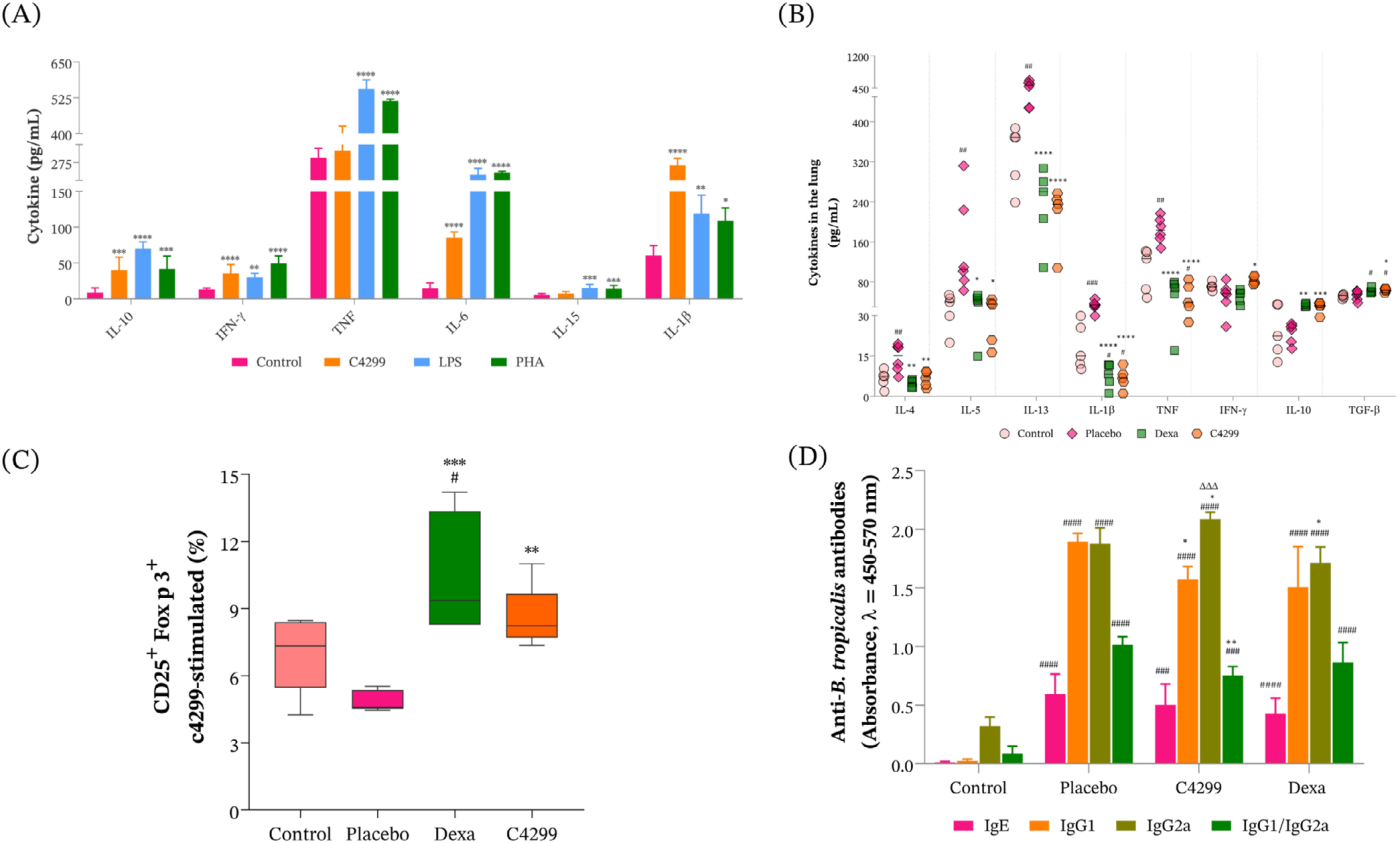
*Recombinant C4299 modulates innate and adaptive immune responses in experimental Blomia tropicalis allergy*. (A) Cytokine secretion by THP‐1‐derived macrophages after stimulation with recombinant C4299. Levels of IL‐10, IFN‐γ, TNF, IL‐6, IL‐15 and IL‐1β were measured by ELISA in culture supernatants and compared with control. LPS and PHA stimuli were used as cell culture controls. (B) Cytokine presence in lung homogenates. (C) Frequency of CD25^+^Foxp3^+^ regulatory T cells among CD4^+^ T cells in splenocytes from treated mice following restimulation with *Blomia tropicalis* extract or C4299 (D) Levels of *B. tropicalis* ‐specific antibodies (IgE, IgG1 and IgG2a) measured in serum from control, placebo‐treated allergic mice, dexamethasone‐treated mice and C4299‐treated mice. Antibody levels were determined by ELISA and expressed as absorbance (450–570 nm). Data are presented as mean ± SD. Statistical significance was determined using one‐way ANOVA followed by Tukey's post hoc test. #Comparison with control. *Comparisons with Sham. Δ Comparisons with Dexa. *, #, Δ *p* ≤ 0.05; **, ##, ΔΔ *p* < 0.01; ***, ###, ΔΔΔ *p* < 0.001. ****, ####, ΔΔΔΔ *p* < 0.0001.

To evaluate its effects in vivo, A/J mice were sensitized and challenged with *B. tropicalis* extract and treated with the recombinant C4299, placebo (only phosphate‐buffered saline) or dexamethasone via subcutaneous injections [5, 6, 7]. In the lungs, C4299 significantly reduced Th2‐associated cytokines (IL‐4, IL‐5 and IL‐13) and similarly decreased pro‐inflammatory cytokines (TNF and IL‐1β), while increasing IL‐10 and TGF‐β levels (Figure 1B). Cytokine levels were also quantified in BAL fluid, confirming the local immunomodulatory effects of C4299. Considering that regulatory T cells are central mediators of immune tolerance, we next examined splenic regulatory responses. Flow cytometric analysis revealed that splenocytes from C4299‐treated mice displayed increased frequencies of CD4^+^CD25^+^Foxp3^+^ regulatory T cells following restimulation with C4299 (Figure 1C). This increase reached levels comparable to those observed with dexamethasone (Figure 1C) and was more pronounced after restimulation with the recombinant protein than with *B. tropicalis* extract, suggesting an antigen‐responsive regulatory mechanism. Consistently, antibodies responses have been also selectively modulated. Treatment with C4299 significantly decreased IgG1 and increased IgG2a levels, resulting in an elevated IgG2a/IgG1 ratio, but IgE levels against *Blomia tropicalis* remained unchanged (Figure 1D). In fact, this antibody pattern is consistent with immune deviation away from Th2 responses and towards a Th1‐associated profile [3, 5, 8]. Importantly, C4299 appeared to induce a more selective immunoregulatory effect than dexamethasone. Similar immunomodulatory findings were achieved when splenocytes were restimulated with *B. tropicalis* extract. These findings indicate attenuation of local Th2 inflammation accompanied by enhancement of regulatory mediators in the airways, a pattern consistent with immune deviation sought in allergen immunotherapy [5, 8, 9]. Additional findings corroborate the regulatory immune response; analysis of bronchoalveolar lavage (BAL) fluid demonstrated that C4299 significantly reduced total leukocyte infiltration compared with placebo. Although reductions in eosinophil numbers and eosinophil peroxidase activity did not reach statistical significance, cytokine profiles revealed a clear immunomodulatory pattern. Splenocyte cultures restimulated with C4299 produced increased levels of IL‐10, TGF‐β and IFN‐γ while reducing IL‐1β, particularly in cells derived from C4299‐treated animals. Together, these results suggest that C4299 promotes systemic regulatory and Th1‐associated immune pathways [3, 5, 8, 9].

Collectively, these findings identify C4299 as a helminth‐derived recombinant protein with relevant anti‐allergic activity. In this model, C4299 reduced airway leukocyte infiltration, downregulated IL‐5 and IL‐13, increased IL‐10 and TGF‐β locally in the lungs, enhanced antigen‐responsive Foxp3^+^ regulatory T cells and shifted antibody responses towards IgG2a. The concomitant induction of IL‐10, TGF‐β, and IFN‐γ suggests immune rebalancing rather than nonspecific immunosuppression [1, 3, 5].

Although the lack of significant IgE reduction and the use of an acute murine model represent limitations, these results support C4299 as a promising candidate for the development of a helminth‐derived immunomodulatory biopharmaceutical or as a potential adjuvant for allergen‐specific immunotherapy strategies [2, 8].

## Author Contributions

C.S.P. conceived and supervised the study, coordinated the project, secured funding and contributed to writing and revising the manuscript. E.S.S. performed the experimental work, contributed to data curation and analysis and assisted in writing and revising the manuscript. J.C.C.A. participated in investigation, experimental procedures, data collection and manuscript preparation. J.E.A.S., J.L.F.M.S., C.M.O.‐F., L.M.S., R.C.S., S.L.B.S., A.M.S.F., J.V.B.R., P.E.O.C., V.L.M.M. and E.M.M.A.B. contributed to investigation, experimental procedures, data acquisition and manuscript review. L.G.C.P. contributed to methodological supervision, provided resources and critically revised the manuscript. All authors reviewed, approved and agreed to the published version of the manuscript.

## Funding

This work was supported by Conselho Nacional de Desenvolvimento Científico e Tecnológico, 168702/2023‐1, 403336/2021‐0 and Fundação de Amparo à Pesquisa do Estado da Bahia, 1414/2025.

## Ethics Statement

All experimental procedures involving animals were approved by the Institutional Animal Care and Use Committee of the Federal University of Bahia (CEUA‐ICS‐UFBA), under protocol number 8373280920. All procedures followed institutional and national guidelines for the ethical treatment of animals in research.

## Conflicts of Interest

The authors declare no conflicts of interest.

## Data Availability

The data that support the findings of this study are openly available in ZENODO at https://zenodo.org/uploads/20295550, reference number https://doi.org/10.5281/zenodo.20295550.

## References

[1] R. M. Maizels and M. S. HJ , “Regulation of the Host Immune System by Helminth Parasites,” Journal of Allergy and Clinical Immunology 138, no. 3 (2016): 666–675, 10.1016/j.jaci.2016.07.007. PMC5010150 27476889

[2] L. F. Santiago , E. S. da Silva , P. S. dos Santos , et al., “The Proteome of Human Adult Whipworm *Trichuris trichiura*: A Source of Potential Immunomodulatory Molecules,” Acta Tropica 263 (2025): 107566, 10.1016/j.actatropica.2025.107566. 39988282

[3] M. Noval Rivas and T. A. Chatila , “Regulatory T Cells in Allergic Diseases,” Journal of Allergy and Clinical Immunology 138, no. 3 (2016): 639–652, 10.1016/j.jaci.2016.06.003. PMC5023156 27596705

[4] L. N. Santos , E. S. Silva , A. S. Santos , et al., “De Novo Assembly and Characterization of the *Trichuris trichiura* Adult Worm Transcriptome Using Ion Torrent Sequencing,” Acta Tropica 159 (2016): 132–141, 10.1016/j.actatropica.2016.03.036. 27038556

[5] C. L. Alves , L. F. Santiago , M. B. Santana , et al., “Immunomodulatory Properties of *Schistosoma mansoni* Proteins Sm200 and SmKI‐1 in Vitro and in a Murine Model of Allergy to the Mite Blomia Tropicalis,” Molecular Immunology 124 (2020): 91–99, 10.1016/j.molimm.2020.05.011. 32544656

[6] E. S. da Silva , M. B. R. de Santana , E. F. Silveira , et al., “The Hybrid Protein BTH2 Suppresses Allergic Airway Inflammation in a Murine Model of HDM‐Specific Immunotherapy,” Clinical & Experimental Allergy 53, no. 8 (2023): 821–832, 10.1111/cea.14293. 36779555

[7] A. M. Fernandes , E. S. da Silva , E. F. Silveira , et al., “Recombinant T‐Cell Epitope Conjugation: A New Approach for *Dermatophagoides* Hypoallergen Design,” Clinical and Experimental Allergy 53, no. 2 (2023): 198–209, 10.1111/cea.14238. 36176209

[8] S. P. Lee , Y. S. Shin , S. Y. Kang , T. B. Kim , and S. M. Lee , “Recent Advances in Allergen‐Specific Immunotherapy in Humans: A Systematic Review,” Immune Network 22, no. 1 (2022): e12, 10.4110/in.2022.22.e12. PMC8901700 35291653

[9] F. Ebner , M. R. Hepworth , S. Rausch , et al., “Therapeutic Potential of Larval Excretory/Secretory Proteins of the Pig Whipworm *Trichuris suis* in Allergic Disease,” Allergy 69, no. 11 (2014): 1489–1497, 10.1111/all.12496. 25069662

